# Nrf2 is highly expressed in neutrophils, but myeloid cell-derived Nrf2 is dispensable for wound healing in mice

**DOI:** 10.1371/journal.pone.0187162

**Published:** 2017-10-26

**Authors:** Natasha Joshi, Sabine Werner

**Affiliations:** Department of Biology, Institute of Molecular Health Sciences, Swiss Federal Institute of Technology (ETH) Zurich, Otto-Stern-Weg 7, Zurich, Switzerland; National Centre for Scientific Research-Demokritos, GREECE

## Abstract

Immune cells of the myeloid lineage are key players in skin wound healing, since they secrete various cytokines and growth factors that orchestrate the repair process. In addition, they are crucial for the defense against invading pathogens through their capacity to produce high levels of reactive oxygen species (ROS). To limit the toxicity of ROS, cells have developed antioxidant defense strategies, including expression of the cytoprotective NRF2 transcription factor. Here we show that murine neutrophils and to a lesser extent macrophages strongly express Nrf2 already when present in the circulation and in particular at the wound site. To determine the role of Nrf2 in neutrophils and macrophages for wound repair, we generated mice with a gain- or loss-of-function of this transcription factor in the myeloid cell lineage. Expression of a constitutively active Nrf2 mutant in myeloid cells did not further enhance the overall Nrf2 activity in these cells due to the already high steady-state activity of endogenous Nrf2. Surprisingly, deletion of Nrf2 in myeloid cells only mildly affected the levels of ROS and the expression of pro-inflammatory cytokines by these cells. In particular, various parameters of wound healing, including wound closure, reepithelialization, wound contraction and the presence of myeloid cells at the wound site were not affected. These results reveal that Nrf2 in myeloid cells is dispensable for wound healing and suggest the presence of additional antioxidant defense strategies of these cells that compensate for the loss of Nrf2, even in the harsh environment of skin wounds.

## Introduction

Wounding of the skin initiates a complex repair process that encompasses several temporally overlapping and highly coordinated events, including blood clotting, inflammation, new tissue formation and finally extensive tissue remodeling. These processes involve interaction among different cell types, including skin resident cells like keratinocytes, fibroblasts and endothelial cells, as well as infiltrating immune cells [1, 2]. Unfortunately, repair of skin wounds in adult mammals is imperfect and results in scar formation and consequent functional impairments. In addition, aged individuals as well as patients treated with anti-inflammatory steroids, chemo- or radiotherapy, and those suffering form certain major diseases, e.g. diabetes and cancer, frequently suffer from impaired healing [2]. This may result in the formation of chronic, non-healing ulcers, which strongly impair the quality of life and cause an enormous financial burden to the health care system [3]. Therefore, there is a strong need to improve the healing process and this requires a thorough understanding of the underlying cellular and molecular mechanims.

Myeloid cells, which include neutrophils, monocytes and macrophages, play important roles during different phases of wound healing, in particular during the early inflammatory phase. These cells produce growth factors and enzmyes that directly contribute to the healing response, together with large amounts of ROS, which are required for the defense against invading pathogens [4]. While this effect of ROS is beneficial, it has been suggested that excessive levels of ROS produced in skin wounds cause tissue damage, delay the healing response, and favour the development of non-healing ulcers and of hypertrophic scars [5]. Therefore, it is important to tightly control the levels of ROS at the wound site. A key player in the control of the cellular ROS balance is the nuclear factor erythroid derived 2-related factor 2 (NRF2; NFE2L2) transcription factor. Under normal conditions the NRF2 inhibitor KEAP1 retains NRF2 in the cytoplasm and also mediates its ubiquitination and subsequent proteasomal degradation. Activation of NRF2 occurs through conformational changes of KEAP1, which are induced by covalent coupling of electrophilic compounds to this protein. These conformational changes result in the weakening of the NRF2-KEAP1 interaction and stabilization of NRF2. Furthermore, it has been suggested that ROS activate certain kinases that phosphorylate NRF2, thereby affecting its binding to KEAP1. As a consequence of these events, the NRF2 turnover is modified and newly synthetized NRF2 is stabilized and accumulates in the nucleus. Upon dimerization with small Maf proteins, NRF2 binds to antioxidant response elements (AREs) in the promoters or enhancers of NRF2 target genes to activate their expression [6, 7]. Among the major NRF2 targets are genes encoding ROS-detoxifying enzymes and other antioxidant proteins, as well as different transporters and proteins involved in the production of the low molecular weight antioxidant glutathione [6].

We and others recently identified important roles of Nrf2 in wound repair of mice. While wound closure in normal mice was not affected in the absence of Nrf2, Nrf2 knockout mice showed a prolonged inflammatory response after wounding, with macrophages still being abundant during the tissue remodelling phase [8]. However, upon induction of diabetes in mice with a global Nrf2 knockout, the wound healing process was significantly delayed compared to diabetic wild-type controls [9]. Conversely, pharmacological activation of Nrf2 or treatment of wounds from diabetic mice with Keap1 siRNA promoted wound healing in diabetic mice [9–11]. It is unclear, however, which cell types require normal Nrf2 levels for efficient wound healing and which cell types would benefit from an increase in Nrf2 activity. In this study we addressed this question and determined the effect of Nrf2 activation and deletion specifically in myeloid cells for wound repair in mice.

## Materials and methods

### Genetically modified mice

CMVcaNrf2 mice have been described previously [12, 13]. By crossing CMVcaNrf2 mice (FVB/N1 genetic background) with LysM-Cre transgenic mice (C57BL/6 genetic background), constitutive expression of the caNrf2 mutant was induced in cells of the myeloid lineage. Mice lacking Nrf2 in myeloid cells were obtained by mating of LysM-Cre transgenic mice with mice carrying floxed alleles for *Nrf2* (C57BL/6 genetic background) [14]. Mice were housed under optimal hygiene conditions and received food and water *ad libitum*. Mouse maintenance and all animal experiments had been approved by the local veterinary authorities of Zurich, Switzerland (Kantonales Veterinäramt Zürich).

### Wounding and preparation of wound tissue

Female mice (8–10 weeks old) were anaesthetized by inhalation of isoflurane prior to and during wound surgery. Four full-thickness excisional wounds of 5 mm diameter were generated on the back of mice using disposable 5mm biopsy punches (Stiefel Laboratorium, Winterthur, Switzerland). Wounds were allowed to heal without dressing. After surgery and during the healing phase, mice were closely observed for any signs of pain or discomfort. In very rare cases where symptoms were observed the mice were sacrificed by CO_2_ inhalation.

For the analysis of the wound healing process, mice were sacrificed by CO_2_ inhalation at different time points after injury, and wounds were excised with a 5mm biopsy punch. Two of the four wounds were placed in RPMI 1640/HEPES medium for analytical flow cytometric analysis. The remaining two wounds were bisected and one half of each wound was snap frozen for RNA isolation and the other half was prepared for histological/morphometric analysis as described below. For fluorescence-activated cell sorting (FACS) experiments we used all the four wounds to obtain enough isolated cells.

We used 3–4 mice per time point for FACS sorting, 3–6 mice (5–12 wounds) per time point and genotype for the analytical flow cytometry analysis and 4–5 mice (6–8 wounds) per genotype for the morphometric analysis of the wounds.

### Histology

For histological analysis of the excised wounds and normal skin, the tissue was fixed overnight in 4% paraformaldehyde (PFA) in PBS or in 95% ethanol / 1% acetic acid, followed by paraffin embedding. Paraffin sections (7μm) from the middle of the wounds were stained using the Herovici procedure [15]. Sections from normal skin were stained with hematoxylin/eosin (H/E). Stained sections were photographed using a Zeiss Axioskop 2 microscope with an Axiocam HRc camera or with the Panoramic 250 Slide Scanner (3D Histech, Budapest, Hungary), and histomorphometrical measurements were quantified using FIJI software [16].

### Isolation of RNA from mouse wounds and from sorted cells

Total RNA was isolated from skin wounds using the RNeasy fibrous tissue mini kit, including a proteinase K digestion step and on-column DNase treatment (Qiagen, Hilden, Germany).

For RNA isolation from sorted cells, they were collected in Eppendorf tubes containing flow buffer (0.2%BSA, 5 mM EDTA in 1 x PBS) supplemented with RNAsin Plus RNase inhibitor (Promega, Madison, WI). The sorted cells were immediately centrifuged, and RNA was isolated using the RNease micro kit with on-column DNase treatment.

### RT-qPCR

cDNA was synthesized using the iScript kit (Bio-Rad Laboratories, Hercules, CA). Quantitative real-time RT-PCR (RT-qPCR) was performed according to the manual of the Light Cycler 480 II (Roche, Rotkreuz, Switzerland). After an initial denaturation step at 95°C for 10min the reaction was performed in 50 cycles (95°C for 10s, 60°C for 20s and 72°C for 20s for each cycle). Samples were analyzed in duplicates. *Rps29* was used as a reference gene. Target gene expression levels were quantified by second derivative method. To determine relative expression levels in the FACS-sorted cells the value from a lymphocyte RNA sample was arbitrarily set to 1 and all the values of the other samples are given relative to this lymphocyte sample. When lymphocytes were not included, a sample from the macrophage population was set to 1. For the RNA data from wound samples, the value from one control mouse was set to 1.

Primers used for RT-qPCR are listed below in Table 1.

**Table 1:** RT-qPCR primer list.

Target gene	Forward sequence 5'->3'	Reverse sequence 5'->3'
*Nrf2*	5’-CCAGCTACTCCCAGGTTGC-3’	5’-CCAAACTTGCTCCATGTCCT-3’
*caNrf2*	5’-TGTTCCTTGTTCCCAAAAGC-3’	5’-CCGGAATATTAATAGGGACGGTA-3’
*Nrf2 Exon5*	5’- TCCATTCCCGAATTACAGTGTCTTA-3’	5’-CGCCAAAATCTGTGTTTAAGGTG-3’
*Nqo1*	5’- TTTGAGAGAGTGCTCGTAGC-3’	5’- GGTCTTCTTATTCTGGAAAGG-3’
*Gclc*	5’-AACAAGAAACATCCGGCATC-3’	5’-CGTAGCCTCGGTAAAATGGA-3’
*Srxn1*	5’-CGGTGCACAACGTACCAAT-3’	5’-TTGATCCAGAGGACGTCGAT-3’
*Slpi*	5’-GGGCAAATACAAGTGCTGTG-3’	5’-CCTGGGAGCAGGGAAGTAGT-3’
*Il1b*	5’-GGACAGAATATCAACCAACAAGTG-3’	5’-TGCTGATGTACCAGTTGGGG-3’
*Il6*	5’-CCGGAGAGGAGACTTCACAG-3’	5’-TTCTGCAAGTGCATCATCGT-3’
*Tnfa*	5’-GACCCTCACACTCAGATCATCTTCT-3’	5’-CCACTTGGTGGTTTGCTACGA-3’
*Vegfa*	5’-GTACCTCCACCATGCCAAGT-3’	5’-CTGCATGGTGATGTTGCTCT-3’
*Rps29*	5'-GGTCACCAGCAGCTCTACTG-3'	5'-GTCCAACTTAATGAAGCCTATGTCC-3'

### Isolation of cells from normal and wounded skin for flow cytometry

Mice were sacrificed, shaved, and the non-wounded back skin or wound biopsies were harvested. In case of normal back skin, the subcutaneous fat was scraped off, and epidermis and dermis were separated using 0.25% dispase (Gibco, Zug, Switzerland) for 50min at 37°C under continuous shaking. The epidermis was treated with 100 KUnits/ml DNase (Sigma) in RPMI supplemented with penicillin/streptomycin (P/S), 10% fetal bovine serum and 20mM HEPES for 45 min at 37°C under continuous shaking. The dermis as well as the wound biopsies were cut into small pieces and treated with 0.25mg/ml TL Liberase (Roche) in RPMI supplemented with P/S and 20 mM HEPES for 1h at 37°C under continuous shaking. Finally, single cell suspensions were prepared by straining the mixture through 70 μm cell strainers, and cells were washed with 1x PBS.

### Flow cytometry

Unspecific binding sites were blocked with anti-CD16/CD32 antibody. Dead cells were labeled with Zombie Aqua™ dye (BioLegend, San Diego, CA) and gated out. Expression of different antigens was analyzed using BD LSRII Fortessa equipped with BD FACSDiva^TM^ 6.0 software (both from BD Biosciences, San Jose, CA). Data analysis was performed using FlowJo v8.7 or FlowJo v10 software (FlowJo, Ashland, OR). Compensation was performed using single color controls prepared from compensation beads (BD Biosciences) and skin samples. Samples were acquired using Fortessa’s High Throughput Sampler plate reader option with an event rate below 20,000 events/s. Compensation matrices calculated by BD FACSDiva^TM^ 6.0 software were applied and cell populations were defined by manual gating. Doublets and dead cells were excluded from the analysis. The dyes and antibodies used for flow cytometry are listed below in Table 2:

**Table 2:** Antibodies and reagents used for flow cytometry.

Antigen	Clone	Fluorophore	Dilution	Source
CD16/32	2.4G2		1:200	BD Biosciences, Franklin Lakes, NJ
CD45	30-F11	BV 605	1:500	BD Biosciences
	30-F11	PB	1:400	BD Biosciences
CD11b	M1/70	PE	1:200	BD Biosciences
	M1/70	BV 711	1:500	BioLegend, San Diego, CA
CD3	17A2	BV 785	1:300	BioLegend
	145-2C11	PE	1:400	BD Biosciences
F4/80	Cl:A3-1	AF 647	1:200	AbD Serotec, Kidlington, UK
Ly6C	AL-21	PE	1:200	BD Biosciences
	HK1.4	PE-Cy7	1:500	BioLegend
	HK1.4	PerCP-Cy5.5	1:500	BioLegend
Ly6G	1A8-Ly6g	AF 700	1:100	BioLegend
	1A8-Ly6g	FITC	1:200	BD Biosciences
	1A8-Ly6g	PerCP-Cy5.5	1:200	BioLegend
Live/Dead		Zombie Aqua	1:400	BioLegend

### Determination of intracellular ROS levels

Mice were euthanized by CO_2_ inhalation and femurs and tibias were removed. The marrow cavities were flushed with sterile Dulbecco’s Modified eagle Medium (DMEM; Sigma-Aldrich, Saint Louis, MO) and single cells were obtained by filtering through 70 μm cell strainers. Cells were plated at equal numbers and then pre-incubated at 37°C for 15 min with 5 μM dichloro-dihydro-fluorescein diacetate (DCFH-DA) and subsequently incubated with the H_2_O_2_-producing enzyme glucose oxidase (85 mU) along with the flow cytometry antibodies for another 15 min and immediately analyzed by flow cytometry using the BD LSRII Fortessa.

### Statistical analysis

Statistical analyses were performed using PRISM software v7 (Graph pad Software Inc., La Jolla, CA. For normal data sets, Student’s t-test or one-way ANOVA tests were performed, depending on whether two or more than two groups were compared. Tukey’s-corrected multiple comparisons with adjusted P value reporting was applied in the case of one-way ANOVA. For non-normal data sets, pairwise comparisons were performed using the Mann-Whitney U rank test. *P ≤0.05; **P ≤0.01; ***P ≤0.001, **** P ≤0.0001.

## Results

### Nrf2 is strongly expressed in neutrophils in the circulation and in the wound tissue

**Fig 1:**
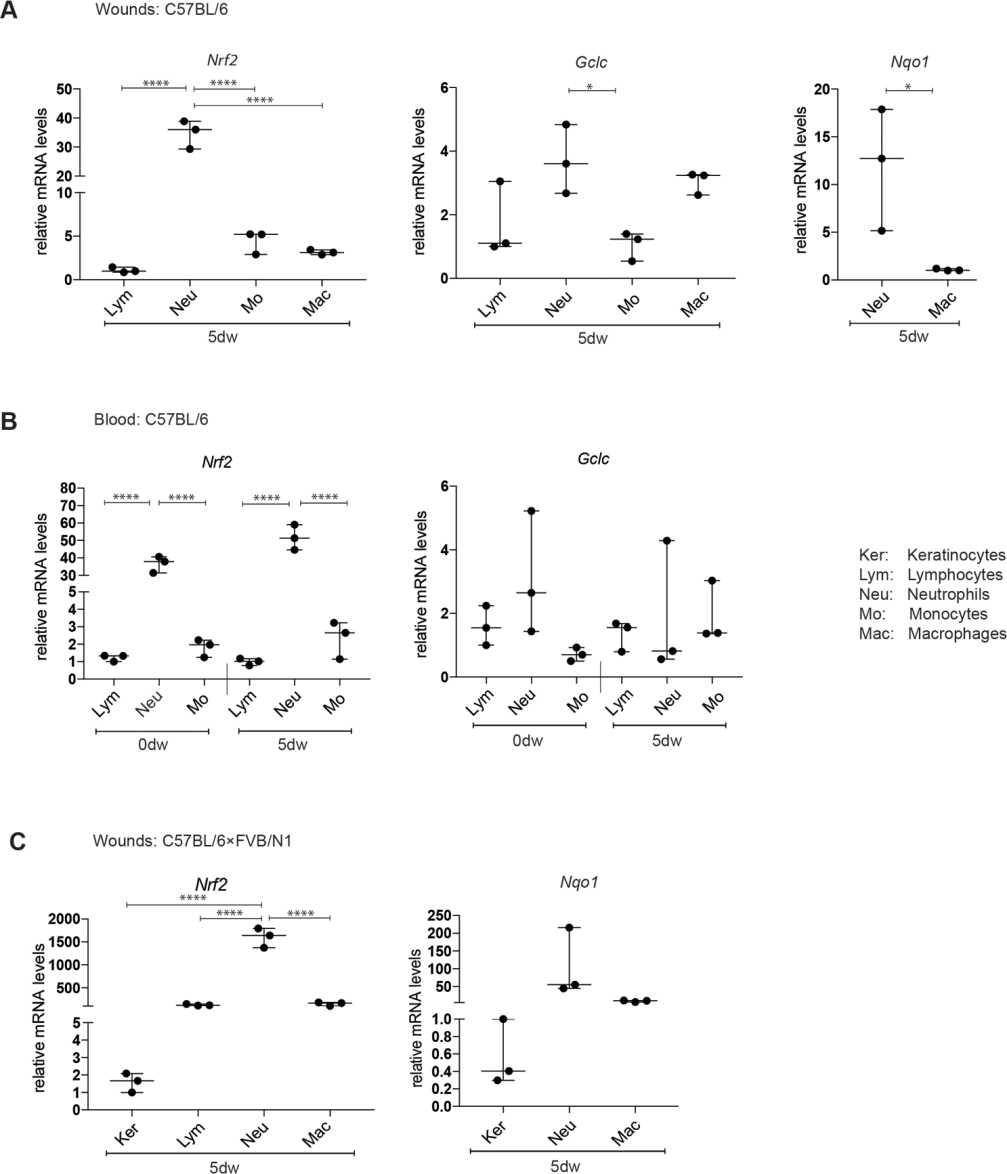
Expression of endogenous Nrf2 and its target genes in keratinocytes and immune cells. Immune cells from the blood or the wound tissue were isolated by FACS based on the following markers: CD45^+^CD11b^-^ (lymphocytes), CD45^+^CD11b^+^Ly6G^+^ (neutrophils), CD45^+^CD11b^+^F4/80^+^ (mainly macrophages, but also some Langerhans cells and monocytes) and CD45^+^CD11b^+^Ly6C^+^ (monocytes/inflammatory macrophages). (A) RT-qPCR analysis using RNA from sorted cells of 5-day wounds (dw) from C57BL/6 mice for *Nrf2*, *Gclc* and *Nqo1* relative to *Rps29*. **(B)** Blood was collected prior to wounding (0dw) and at day 5 after wounding (5dw) of C57BL/6 mice, and RNA from FACS-sorted immune cells was analyzed by RT-qPCR analysis for *Nrf2* and *Gclc*. **(C)** RT-qPCR analysis using RNA from sorted immune cells and keratinocytes (CD49f^+^CD140a^-^CD45^-^CD31^-^) of 5-day wounds (dw) from C57BL/6 x FVB/N1 mice for *Nrf2*, *Gclc* and *Nqo1* relative to *Rps29*. N = 3–6 mice. Bars indicate median with 95% confidence interval (CI). Each data point represents the result from one mouse. *P ≤0.05; **P ≤0.01; ***P ≤0.001, **** P ≤0.0001 (one-way ANOVA for multiple group comparisons and Student’s t-test for two-group comparison).

To investigate the expression of *Nrf2* in myeloid cells during wound healing, we generated full-thickness excisional wounds on the back of wild-type mice of C57BL/6 genetic background and isolated different types of immune cells from the wound tissue at day 5 after injury when neutrophils and macrophages are abundant. For this purpose we established an optimized procedure based on gentle dissociation of the skin samples followed by fluorescence activated cell sorting (FACS) with a pre-established gating strategy (S1 Fig). In the same experiment, we isolated different populations of immune cells from the blood prior to wounding and during the healing phase. The isolated cells included neutrophils (CD45^+^CD11b^+^Ly6G^+^), a macrophage-enriched population (CD45^+^CD11b^+^F4/80^+^ that also includes some Langerhans cells and monocytes [17]), monocytes/inflammatory macrophages (CD45^+^CD11b^+^Ly6C^+^) and lymphocytes (CD45^+^CD11b^-^). Interestingly, Nrf2 mRNA levels were extremely high in neutrophils of the wound tissue and of the blood and more than 10-fold higher compared to monocytes, macrophages or non-myeloid cells (lymphocytes) (Fig 1A and 1B). We also analyzed at least one of the classic Nrf2 target genes NAD(P)H dehydrogenase quinone 1 (*Nqo1*) and glutamate-cysteine ligase catalytic subunit (*Gclc*) [7]. Indeed, the different Nrf2 expression levels between wound neutrophils and macrophages were reflected by similar differences in the expression of *Nqo1*, while the difference between neutrophils and macrophages was less pronounced for *Gclc* (Fig 1A and 1B). Since we subsequently used a C57BL/6 × FVB/N1 background for the generation of *Nrf2* mutant mice, we repeated this experiment in the mixed background and confirmed the strong expression of *Nrf2* and *Nqo1* in wound neutrophils. Remarkably, *Nrf2* mRNA levels were more than 500-fold higher in neutrophils compared to keratinocytes that had been freshly isolated by FACS (CD49f^+^CD140a^-^CD45^-^CD31^-^) (Fig 1C). This is remarkable, since Nrf2 plays an important role in keratinocytes under different conditions [18]. This experiment also revealed that wound lymphocytes express higher levels of Nrf2 compared to keratinocytes, but still much less compared to neutrophils (Fig 1C).

### Genetic activation of Nrf2 in myeloid cells does not affect cutaneous wound healing

**Fig 2:**
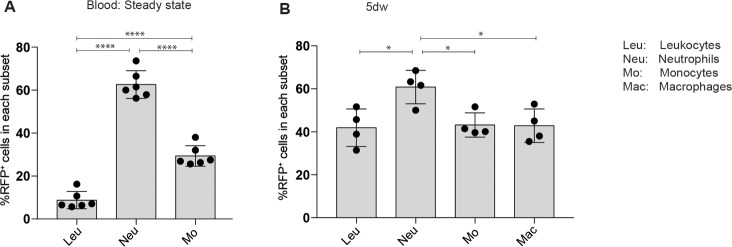
Characterization of deletion efficiency of LysM-Cre mice in blood and wound leukocytes. Flow cytometry analysis to quantify the percentage of red fluorescent protein positive (RFP^+^) cells among all leukocytes or among neutrophils, monocytes or macrophages **(A)** in the blood of non-injured mice (N = 6 mice) or **(B)** in 5-day wounds (n = 4 wounds obtained from two different mice (N = 2)). Bars indicate mean ±SD. *P ≤0.05; **P ≤0.01; ***P ≤0.001, **** P ≤0.0001 (one-way ANOVA).

We next determined if modification of Nrf2 expression in myeloid cells affects the wound healing process. To target these cells we made use of LysM-Cre transgenic mice [19]. To verify the specificity of this promoter for myeloid cells in wound tissue, we used LysM-Cre/ROSA-RFP mice, which express red fluorescence protein (RFP) in cells in which the Lysozyme M promoter is active. Flow cytometry revealed that approximately 60% of the neutrophils were RFP positive in the blood or in 5-day full-thickness excisional wounds, but only 30–40% of the monocytes and macrophages (Fig 2A and 2B and S2 Fig).

**Fig 3:**
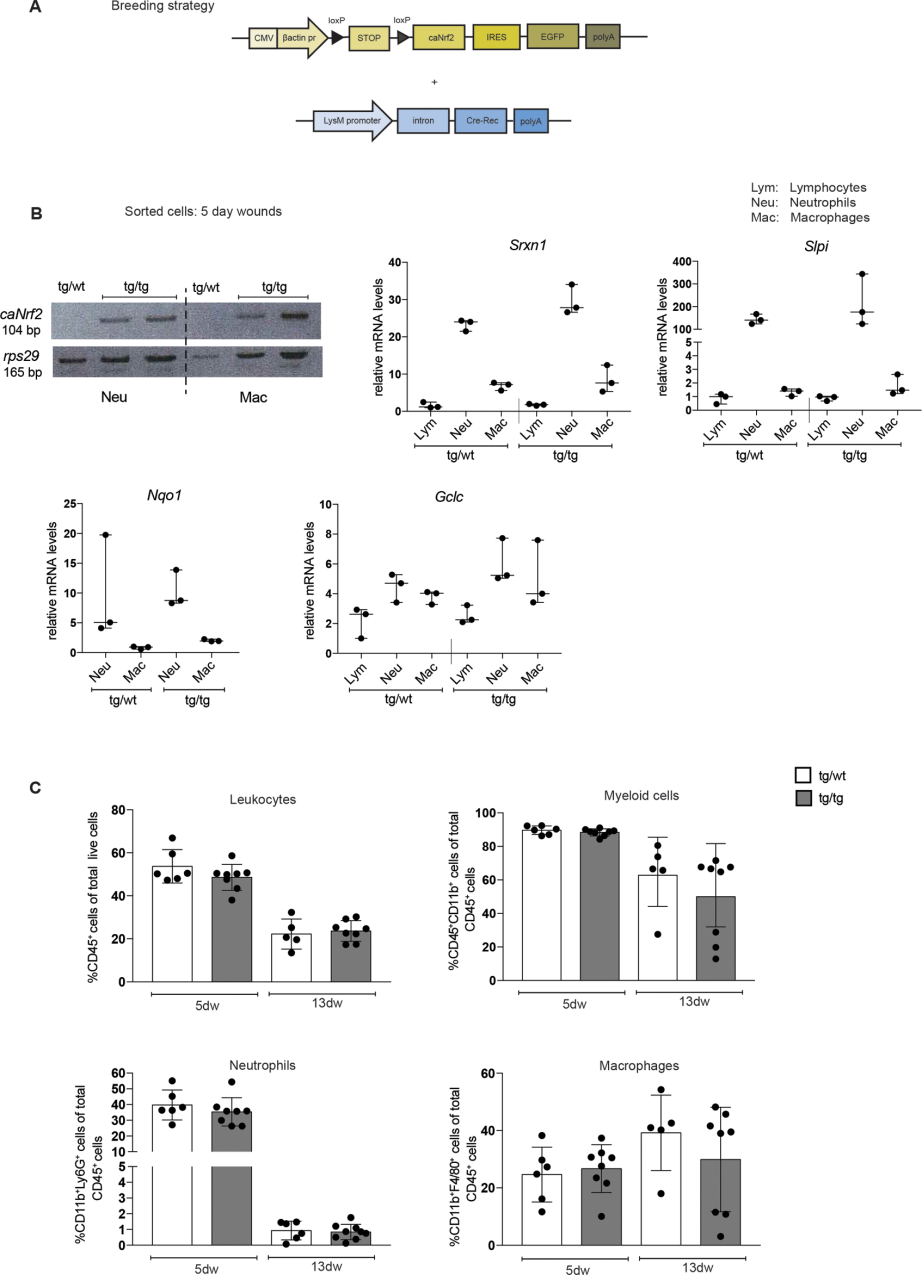
Genetic activation of Nrf2 in myeloid cells. **(A)** Schematic representation of the transgenes used for the generation of LysM-Cre CMVcaNrf2 mice. **(B)** Left upper panel: RNA from sorted neutrophils or macrophages from 5dw of control (tg/wt) and LysM-cre CMVcaNrf2 mice (tg/tg) were analyzed for *caNrf2* transgene expression and for *Rps29* by RT-qPCR. PCR products were analyzed by agarose gel electrophoresis. Other panels: RT-qPCR using RNA from lymphocytes, neutrophils and macrophages isolated from 5d wounds for the Nrf2 target genes *Gclc*, *Srxn1*, *Slpi*, and *Nqo1* relative to *Rps29*. N = 3 mice. Bars indicate median with 95% CI. **(C)** Flow cytometry analysis of 5dw and 13dw of tg/wt and tg/tg mice for total leukocytes, myeloid cells, neutrophils and macrophages. N = 3–5 mice, n = 5–10 wounds. Bars indicate mean ±SD. *P ≤0.05; **P ≤0.01; ***P ≤0.001, **** P ≤0.0001 (Mann-Whitney U test).

Being aware of the only partial targeting of the wound myeloid cell population by the LysM-Cre transgenic mice, we used them to express a constitutively active Nrf2 mutant (caNrf2) in myeloid cells by mating them with mice expressing caNrf2 under control of a cytomegalovirus enhancer and a β-actin promoter. To avoid constitutive expression of the transgene, it is preceded by a transcription/translation STOP cassette flanked by *loxP* sites (Fig 3A) [12]. Upon mating with LysM-Cre mice, myeloid cells are expected to express the *caNrf2* transgene.

The double transgenic mice were viable and fertile and did not reveal obvious macroscopic abnormalities. Histological analysis of their non-injured skin did not uncover obvious abnormalities. Furthermore, flow cytometry analysis of skin immune cells and blood cells did not reveal a significant difference between caNrf2-transgenic mice and their littermate controls (S3 Fig).

To verify the specific expression of the transgene in myeloid cells, we isolated lymphocytes, neutrophils, and macrophages from 5-day wounds of double transgenic LysM-Cre CMV-caNrf2 mice (designated tg/tg) and single transgenic LysM-Cre control mice (designated tg/wt) using FACS. RT-PCR analysis revealed that caNrf2 was indeed only expressed in neutrophils and macrophages of the double mutant mice (Fig 3B). Surprisingly, however, expression of Nrf2 target genes, including *Gclc*, sulfiredoxin-1 (*Srxn1)*, secretory leukocyte protease inhibitor (*Slpi)*, and *Nqo1*, was only slightly, but non-significantly higher in tg/tg mice (Fig 3B). This experiment was performed with mice in a mixed genetic background (C57BL/6 × FVB/N1) and with mice in a pure C57BL/6 background (S4 Fig). Furthermore, flow cytometry revealed no differences in the number of total leukocytes, total myeloid cells, neutrophils and macrophages, in the wounds at day 5 and day 13 after injury (Fig 3C). These findings suggest that the caNrf2 mutant cannot further increase the high activity of Nrf2 in these cells, and we therefore did not further characterize the wound healing process in these animals.

### Generation of mice lacking Nrf2 in myeloid cells

**Fig 4:**
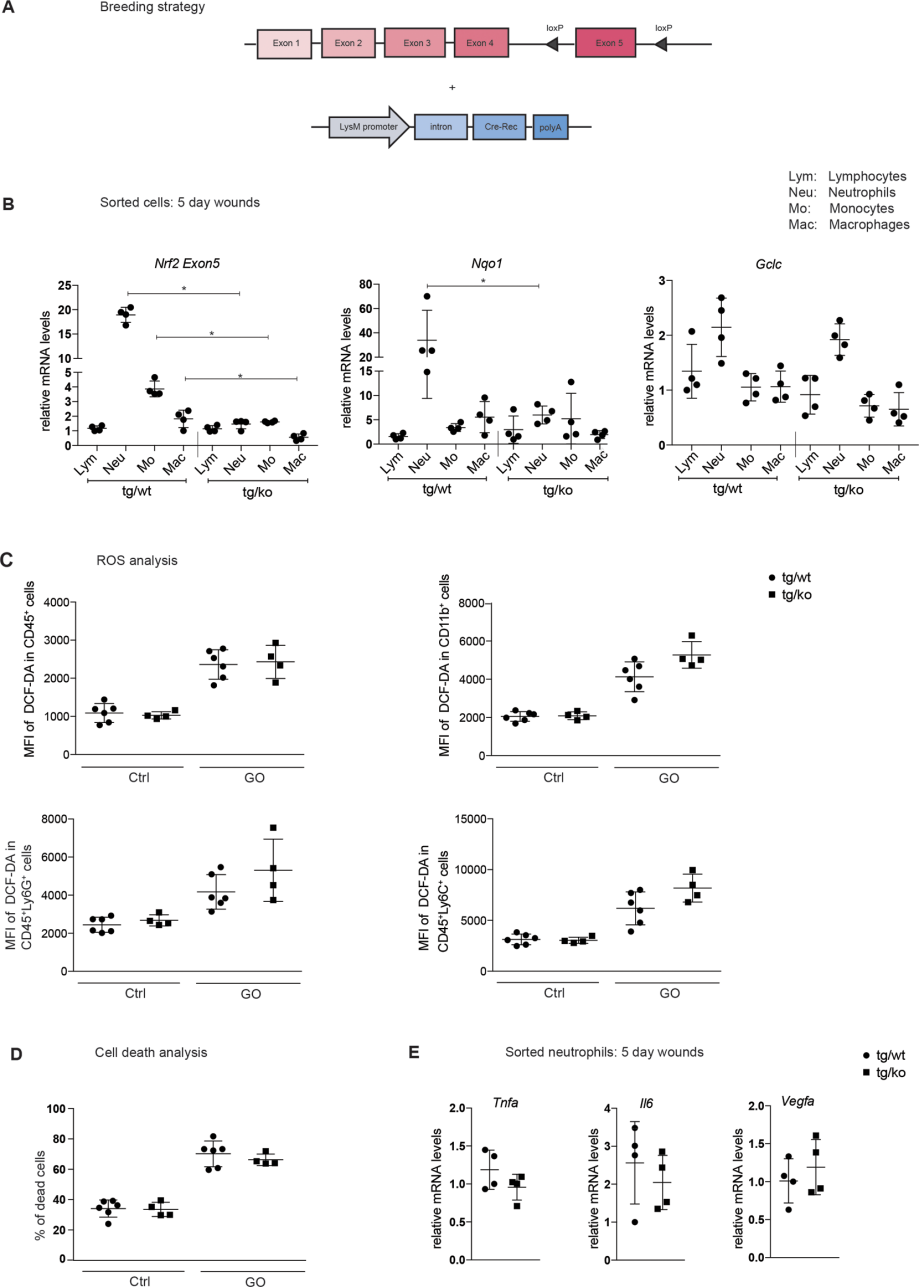
Characterization of LysM-Cre Nrf2-ko mice. **(A)** Schematic representation of the breeding strategy for LysM-Cre Nrf2-ko mice. **(B)** RNA from different immune cells sorted from 5dw of control (tg/wt) and LysM-Cre Nrf2-ko (tg/ko) mice were analyzed by RT-qPCR for *Nrf2* (exon5), *Nqo1* and *Gclc* relative to *Rps29*. N = 4 mice per genotype. *P ≤0.05 (Mann-Whitney U test). **(C)** Bone marrow derived cells (BMDCs) from tg/wt and tg/ko mice were pre-incubated for 15 min with 5 μM DCFH-DA and subsequently stimulated with glucose oxidase (GO; 85 mU) or left untreated (control) for another 15 min. Cells were analyzed by flow cytometry based on DCF fluorescence (reflecting ROS levels). Results shown are representatives of at least three independent experiments. N = 2–3 mice per experiment, two data points represent each mouse. **(D)** Survival of BMDCs after GO treatment. The percentage of dead cells as determined by flow cytometry is shown. **(E)** RNA from sorted neutrophils of 5dw was analyzed by RT-qPCR for *Tnfa*, *Il6* and *Vegfa* relative to *Rps29*. N = 4 mice. Bars indicate mean ±SD. *P ≤0.05; **P ≤0.01; ***P ≤0.001, **** P ≤0.0001 (Mann-Whitney U test).

Due to the strong expression of Nrf2 in myeloid cells, it was particularly interesting to determine the consequences of a loss of Nrf2 in these cells for wound repair. For this purpose, we mated mice with floxed *Nrf2* alleles [14] with LysM-Cre mice (Fig 4A). The double transgenic LysM-Cre-Nrf2-ko mice (designated tg/ko) were viable and fertile, and did not exhibit obvious abnormalities, including no alterations in the histological appearance of the skin. Flow cytometry analysis demonstrated that loss of Nrf2 in myeloid cells did not affect major immune cell populations in the non-injured skin (S5 Fig).

We next isolated different types of immune cells from 5-day wounds by FACS and analyzed the expression of *Nrf2* and selected target genes. As expected, there was a significant reduction in the levels of the full-length *Nrf2* mRNA in neutrophils and to a lesser extent in monocytes and macrophages of tg/ko mice as determined by RT-qPCR with primers for exon 5, which is targeted in the mutant mice. This correlated with significantly reduced expression of *Nqo1*, and a milder reduction of *Gclc* expression was also detectable (Fig 4B). By contrast, and as expected from the LysM promoter activity, expression of *Nrf2* and Nrf2 target genes was not affected in lymphocytes (Fig 4B).

Since the knockout of Nrf2 was particularly efficient in neutrophils, we further analyzed the consequences of the Nrf2 knockout for these cells. Due to the important role of Nrf2 in the control of the cellular redox balance, we treated bone marrow derived cells (BMDC) from tg/ko and tg/wt mice with glucose oxidase, which results in continuous production of hydrogen peroxide. The levels of ROS in these cells were then measured using a 2`,7`- DCFH-DA-based assay. However, the loss of Nrf2 only caused a very mild, but non-significant increase in ROS levels in the different myeloid cell populations (Fig 4C). There was also no difference in the percentage of dead cells before or after GO treatment of the BMDCs (Fig 4D).

Finally, loss of Nrf2 in neutrophils did not affect the expression of the pro-inflammatory cytokines tumor necrosis factor alpha (*Tnfa*) and interleukin 6 (*Il6*) or of the pro-angiogenic factor vascular endothelial growth factor A (*Vegfa*) (Fig 4E).

### Nrf2 in myeloid cells is dispensable for wound healing in mice

**Fig 5:**
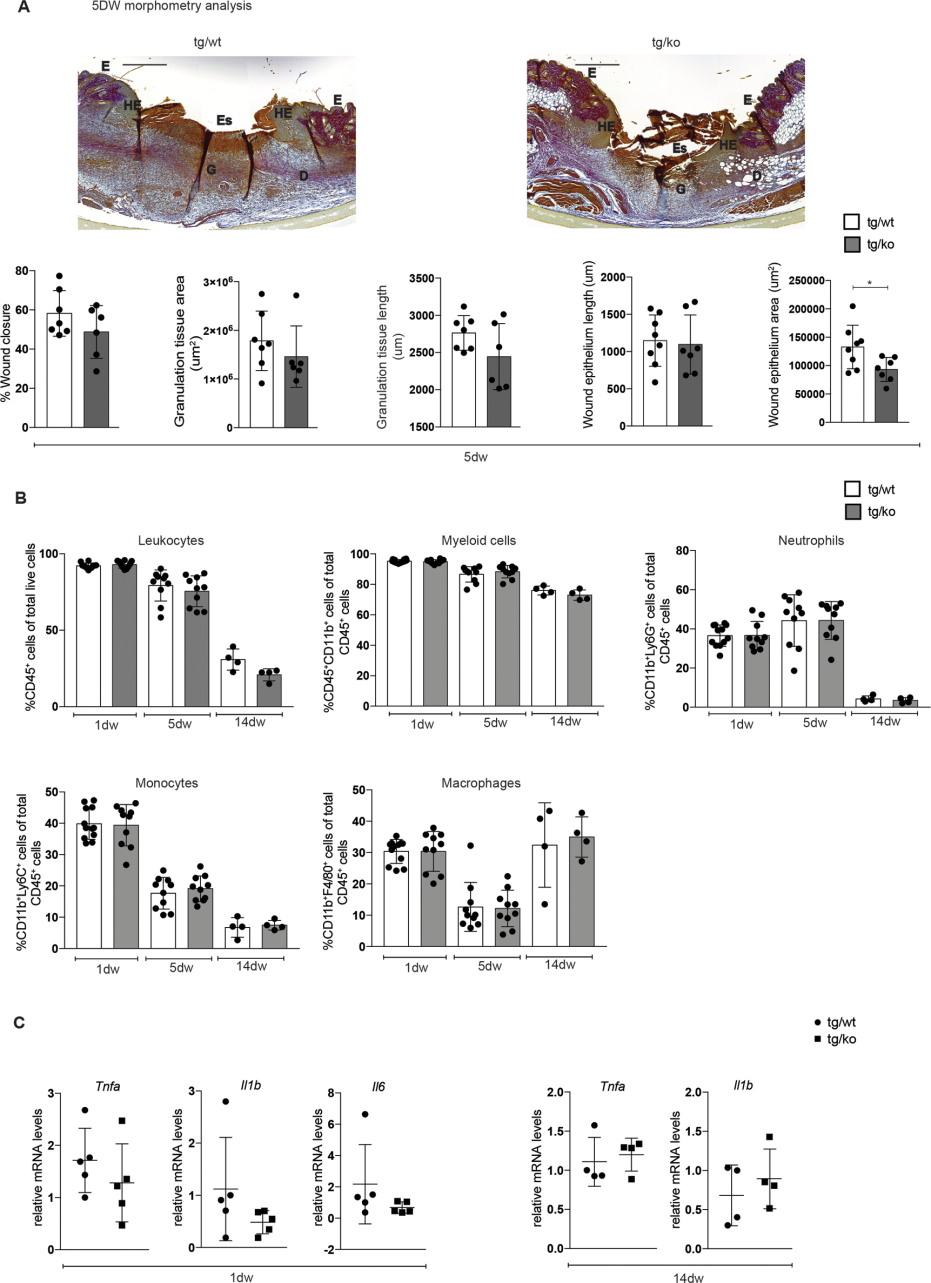
Wound healing is not affected in LysM-Cre Nrf2-ko mice. **(A)** Representative pictures of Herovici-stained sections from 5dw of tg/wt and tg/ko mice. Scale bar: 500 μm. D: Dermis; E: Epidermis, Es: Eschar; G: Granulation tissue; HE: Hyperproliferative wound epidermis. Quantitative data from a morphometric analysis of 5dw of tg/wt and tg/ko mice for wound closure, area and length of the granulation tissue, and area and length of wound epithelium are shown below (N = 3–4 mice, n = 6–8 wounds). The raw data of the morphometric analysis of the wounds are shown in S1 Table. **(B)** Flow cytometry analysis of 1dw, 5dw and 14dw of tg/wt and tg/ko mice for total leukocytes, myeloid cells, neutrophils, monocytes and macrophages. N = 4–6 mice, n = 8–12 wounds. **(C)** RNA from 1dw and 14dw from control and conditional knockout mice was analyzed for *Tnfa*, *Il1b* and *Il6* relative to *Rps29*. Bars indicate mean ±SD. *P ≤0.05; **P ≤0.01; ***P ≤0.001, **** P ≤0.0001 (Mann-Whitney U test).

Since loss of Nrf2 in all cells affected the inflammatory response in healing wounds [8], we investigated if this phenotype results directly from the loss of Nrf2 in myeloid cells. Histomorphometric analysis of sections from the middle of the wounds did not reveal significant alterations in wound closure, granulation tissue area and length, or length of wound epithelium in 5-day wounds of tg/ko mice compared to their littermate controls (Fig 5A). The area of wound epithelium was significantly smaller in the tg/ko compared to control mice, indicating that Nrf2-deficient myeloid cells that are present in the wound bed affect keratinocytes in a paracrine manner. However, the rate of wound closure was not significantly reduced. Furthermore, the immune cell composition at day 1, day 5 and day 14 after wounding was not obviously affected, suggesting that the lack of Nrf2 in myeloid cells neither affected the infiltration of immune cells nor their resolution (Fig 5B). Consistent with this finding, expression of the pro-inflammatory cytokines *Tnfa*, *Il1b* and *Il6*, which are highly expressed in neutrophils early after injury [20], was not affected in 1-day wounds of tg/wt mice, and *Tnfa* and *Il1b* mRNA levels were also similar in 14-day wounds of mice of both genotypes (Fig 5C).

Together, these results show that loss of Nrf2 in myeloid cells does not affect the wound healing capability in normal mice. Therefore, the altered inflammatory response that occurred during wound healing in mice with global Nrf2 knockout is most likely an indirect effect that is mediated via skin resident cells.

## Discussion

Hematopoietic cells of the myeloid lineage play an irreplaceable role in host defense mechanisms. They are also key regulators of wound healing, a process that involves an intricate and highly orchestrated interplay between various innate and adaptive immune cells along with the skin resident epithelial and mesenchymal cells [1, 4, 21]. A major task of myeloid cells in wound healing is the production of high levels of ROS as a mechanism to defend invading pathogens. However, these reactive compounds can severely damage cells in the wound bed, including the myeloid cells themselves. Therefore, these cells had to develop various antioxidant defense strategies, including expression of the cytoprotective transcription factor Nrf2 [22–24]. Indeed, abnormal expression/activation of Nrf2 in myeloid cells had severe effects in different mouse models for major human diseases, including, but not limited to cancer, atherosclerosis, and emphysema [25]. Therefore, we speculated that Nrf2 in myeloid cells has an important role in wound healing.

Consistent with this assumption we show here that myeloid cells, in particular neutrophils, display very high *Nrf2* expression not only during wound healing, but also while circulating in the blood under physiological conditions. This finding suggests that Nrf2 protects these cells from oxidative damage in injured/inflamed tissues and allows them to rapidly adapt to inflammatory conditions. This beneficial activity may be further promoted by activation of endogenous Nrf2, since it has previously been shown in *in vitro* and *ex vivo* settings that chemical activation of Nrf2 in macrophages and neutrophils can have anti-inflammatory or anti-oxidative effects, respectively [26, 27]. On the other hand, loss of Nrf2 in myeloid cells was detrimental in different settings. For example, knockout of Nrf2 in myeloid cells aggravated the symptoms in a mouse model of systemic sepsis and resulted in dramatically reduced survival of the mice, whereas deletion of the Nrf2 antagonist Keap1 in myeloid cells had a protective function in this model. This finding was explained by enhanced ROS detoxification by myeloid cells in the absence of Keap1 [14]. However, Keap1 has also Nrf2-independent functions [28, 29], which may contribute to this phenotype. This limitation can be overcome by the use of a constitutively active Nrf2 mutant. Using this approach, it was shown that genetic activation of Nrf2 in myeloid cells leads to less inflammation and ultimately less tissue injury in the liver after hepatic ischemia/reperfusion injury [30]. Furthermore, genetic activation of Nrf2 in myeloid cells aggravated acute colitis in mice, while it had a beneficial effect in a chronic colitis model [31]. These findings suggest that the effect of Nrf2 activation may depend on the affected organ, the injury model and on the duration of the inflammation.

To our surprise, however, neither Nrf2 activation nor loss of this protein in myeloid cells affected the wound healing process in mice. The lack of an effect of Nrf2 activation may be explained by the already high expression and activity of this transcription factor in myeloid cells of the wound tissue, which could not be further enhanced by the caNrf2 mutant. This may be different in myeloid cells of the liver or intestine. Alternatively, caNrf2 may be able to upregulate other Nrf2 target genes that we have not analyzed and which may be important in repair of the liver and intestine, but not in murine wound repair. Finally, it may well be that the targeting by the LysM promoter is more efficient in myeloid cells of the liver and intestine compared to wound myeloid cells. The rather inefficient targeting of myeloid cells, in particular of macrophages, may explain at least in part why we did not observe an effect on wound repair in mice lacking Nrf2 in myeloid cells. Thus, myeloid cells with wild-type Nrf2 may still be present in the wound at significant levels and they could be sufficient to allow normal healing. However, the very low levels of Nrf2 that we detected in isolated wound neutrophils of the knockout mice suggest that at least Nrf2-deficient neutrophils are not out-competed by cells that have escaped recombination.

We had previously shown a prolonged presence of macrophages in wounds of mice with a global Nrf2 knockout [8]. Surprisingly, this was not the case in mice lacking Nrf2 only in myeloid cells, suggesting that expression of Nrf2 in other skin cell types indirectly affects the duration of the inflammatory phase during wound healing. Consistent with this hypothesis, Nrf2 from non-myeloid cells contributed to high fat diet-induced tissue inflammation and insulin resistance, whereas loss of myeloid-derived Nrf2 had no effect [32].

While loss of Nrf2 in myeloid cells and even in all cells is obviously dispensable for wound healing in normal mice, Nrf2 seems to be crucial for wound healing in diabetic mice, which are characterized by impaired healing and enhanced oxidative stress [33]. Thus, treatment of wounds in diabetic mice with siRNA against the Nrf2 antagonist Keap1 reduced the oxidative stress and hence, resulted in accelerated wound closure [10, 11]. Vice versa, diabetic mice with global Nrf2 knockout showed delayed wound healing compared to their wild-type littermates as a consequence of more severe oxidative DNA damage, reduced cell proliferation and migration and increased apoptosis [9]. Therefore, it may well be that loss of Nrf2 in myeloid cells also aggravates the wound healing defect in diabetic mice and this should be addressed in future studies. The normal healing in healthy mice lacking Nrf2 in myeloid cells suggests, however, that these cells have developed additional and potent antioxidant defense mechanisms that are not dependent on Nrf2. Such a redundancy may be required for their survival under various harsh conditions, such as in a wound environment. The identification of these additional mechanisms will be an important goal for the future.

## Supporting information

S1 Fig Gating strategy for sorting cells from 5-day wounds. Representative flow cytometry gating plots for sorting different populations of immune cells from 5dw. The live single cells were selected for myeloid cells (CD45^+^CD11b^+^) and these cells were further gated for different myeloid cell subtypes, including neutrophils, macrophages and monocytes. All gates are based on fluorescence minus one control.
(PDF)

S2 Fig Gating strategy for characterizing LysM-Cre/Rosa-RFP mice. Representative pseudocolor plots for the gating strategy to quantify the percentage of RFP-positive cells among different immune cell populations of control and transgenic mice in **(A)** blood in the steady state and in **(B)** 5dw.
(PDF)

S3 Fig Characterization of non-wounded skin from LysM-Cre-CMVcaNrf2 mice. **(A)** Representative pictures of H/E-stained sections from the back skin of 10-week-old female mice. Scale bar: 200 μm. **(B)** Flow cytometry analysis of dissociated single cells from the back skin epidermis or dermis or from the blood of tg/wt and tg/tg mice for the quantification of different immune cell populations as indicated. Bars indicate mean ±SD.
(PDF)

S4 Fig Genetic activation of Nrf2 in myeloid cells of C57BL/6 mice. **(A)** RT-qPCR using RNA from lymphocytes, neutrophils and macrophages isolated from 5d wounds for the classical Nrf2 target genes *Gclc* and *Nqo1* relative to *Rps29*. N = 3 mice. Bars indicate median with 95% CI.
(PDF)

S5 Fig Characterization of non-wounded skin from LysM-Cre-Nrf2-ko mice. **(A)** Representative pictures of H/E- stained sections from the back skin of 10-week-old female mice. Scale bar: 200 μm. **(B)** Flow cytometry analysis of dissociated single cells from the back skin epidermis or dermis of tg/wt and tg/ko mice for the quantification of different immune cell populations as indicated. Bars indicate mean ±SD.
(PDF)

S1 Table Raw data of the morphometric analysis of wounds from LysM-Cre-Nrf2-ko and ctr. mice. (XLSX)
