# Supplementary figures and images for: Nrf2 is highly expressed in neutrophils, but myeloid cell-derived Nrf2 is dispensable for wound healing in mice
Source: PLoS One. 2017 Oct 26;12(10):e0187162. doi: 10.1371/journal.pone.0187162 (PMC5658185; doi:10.1371/journal.pone.0187162)

S1 Fig. Gating strategy for sorting cells from 5-day wounds.

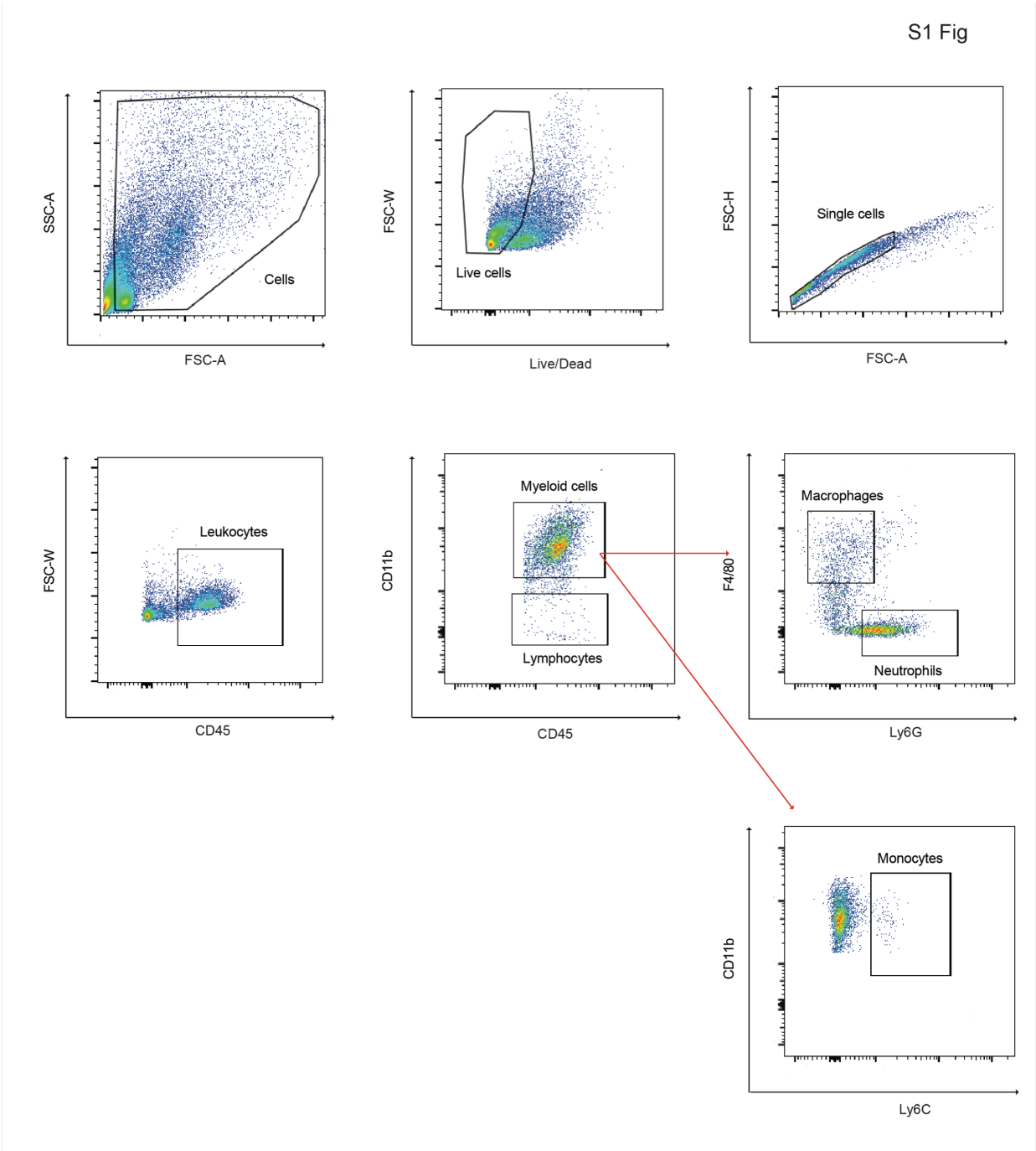

Supplement: S1 Fig — Representative flow cytometry gating plots for sorting different populations of immune cells from 5dw. The live single cells were selected for myeloid cells (CD45+CD11b+) and these cells were further gated for different myeloid cell subtypes, including neutrophils, macrophages and monocytes. All gates are based on fluorescence minus one control. (PDF) [file pone.0187162.s001.pdf]

S2 Fig. Gating strategy for characterizing LysM-Cre/Rosa-RFP mice.

S2 Fig

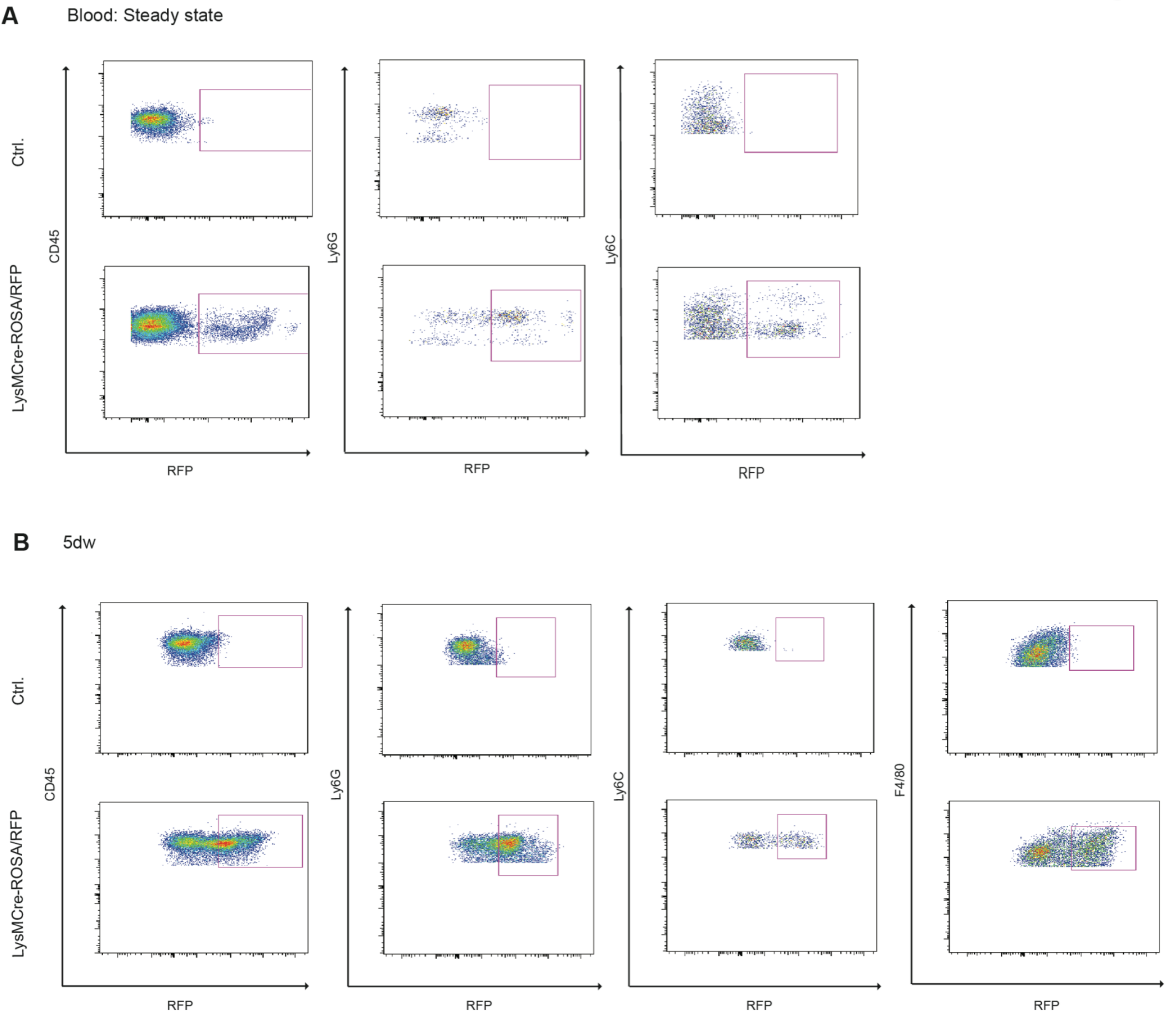

Supplement: S2 Fig — Representative pseudocolor plots for the gating strategy to quantify the percentage of RFP-positive cells among different immune cell populations of control and transgenic mice in (A) blood in the steady state and in (B) 5dw. (PDF) [file pone.0187162.s002.pdf]

S3 Fig. Characterization of non-wounded skin from LysM-Cre-CMVcaNrf2 mice.

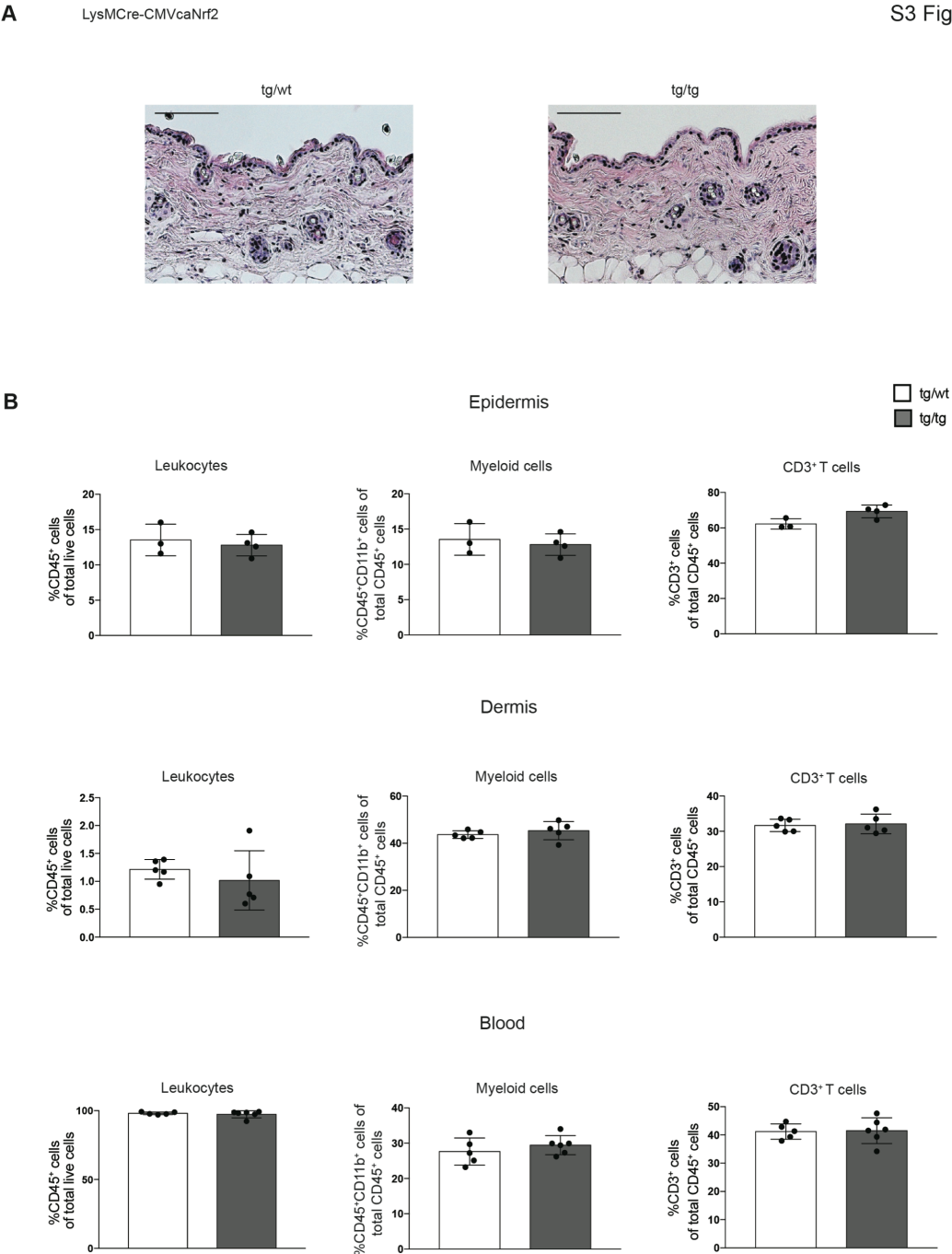

Supplement: S3 Fig — (A) Representative pictures of H/E-stained sections from the back skin of 10-week-old female mice. Scale bar: 200 μm. (B) Flow cytometry analysis of dissociated single cells from the back skin epidermis or dermis or from the blood of tg/wt and tg/tg mice for the quantification of different immune cell populations as indicated. Bars indicate mean ±SD. (PDF) [file pone.0187162.s003.pdf]

S4 Fig. Genetic activation of Nrf2 in myeloid cells of C57BL/6 mice.

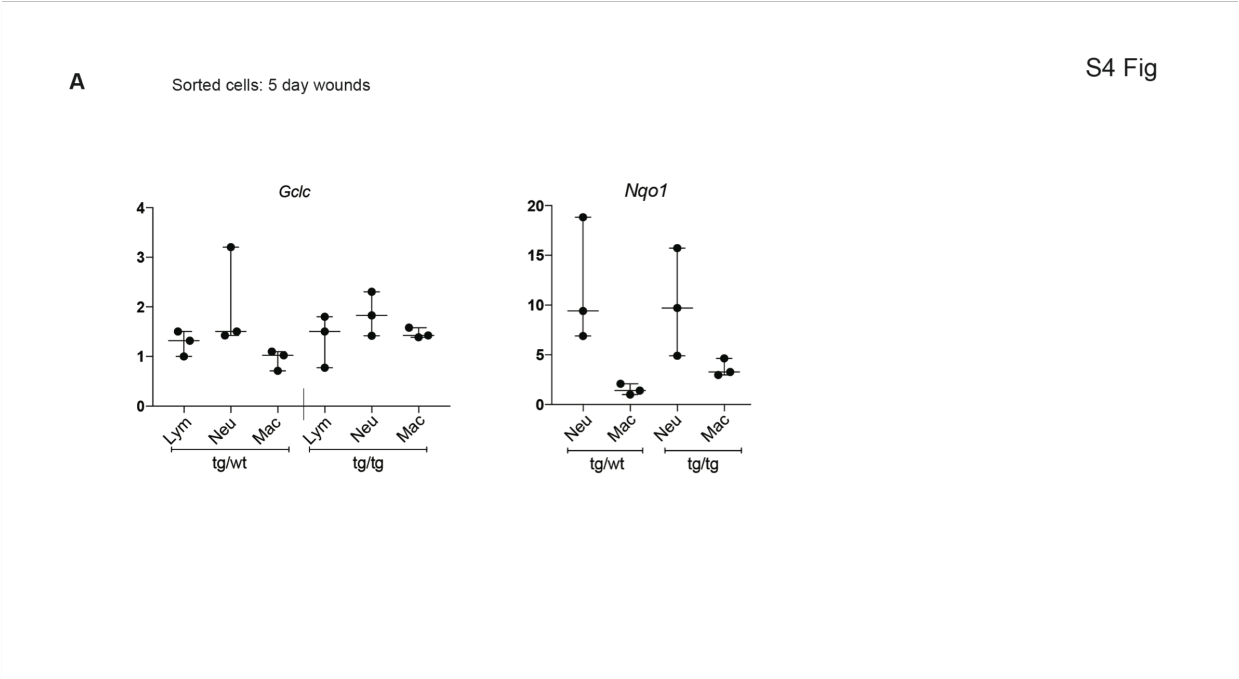

Supplement: S4 Fig — (A) RT-qPCR using RNA from lymphocytes, neutrophils and macrophages isolated from 5d wounds for the classical Nrf2 target genes Gclc and Nqo1 relative to Rps29. N = 3 mice. Bars indicate median with 95% CI. (PDF) [file pone.0187162.s004.pdf]

S5 Fig. Characterization of non-wounded skin from LysM-Cre Nrf2-ko mice.

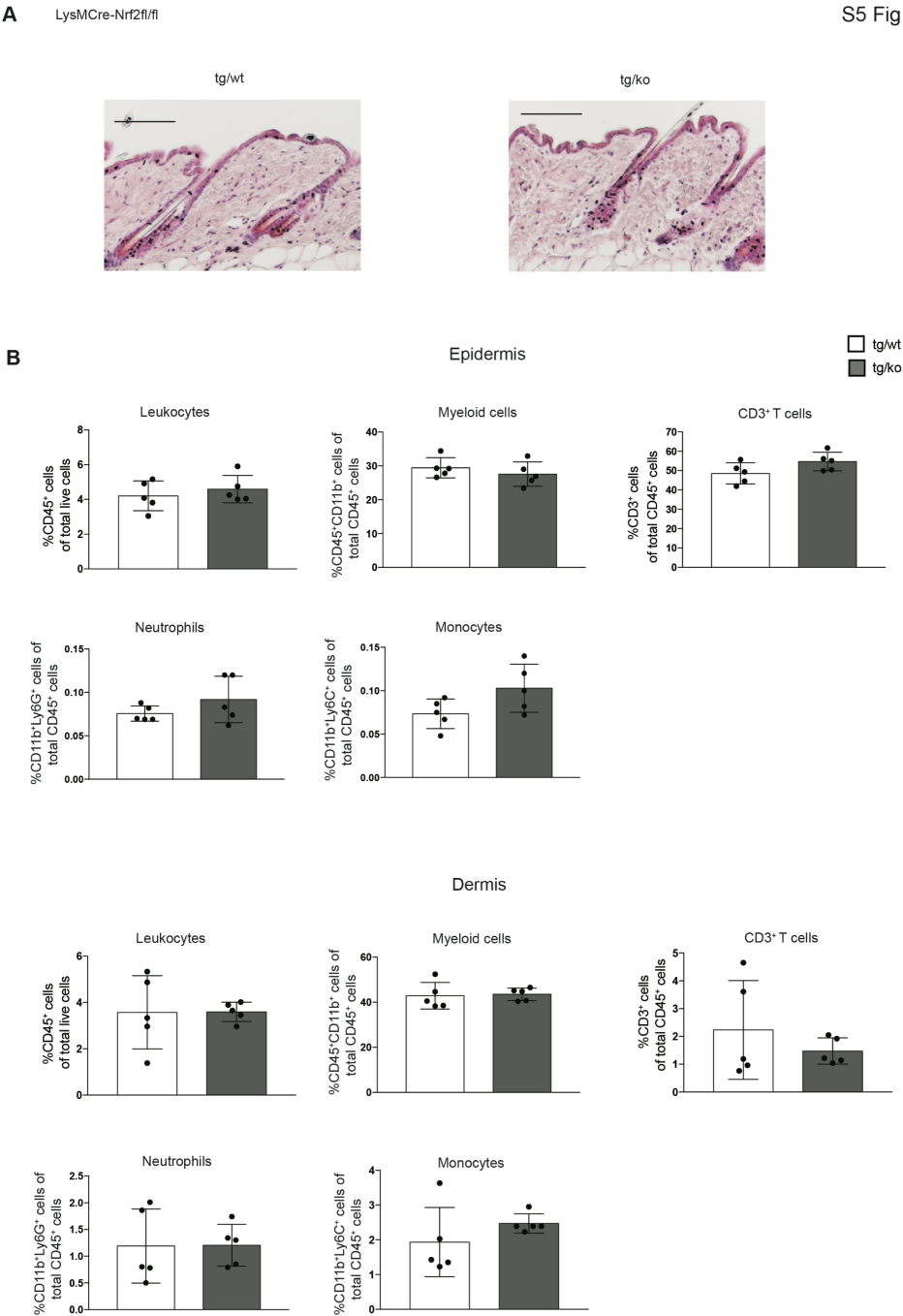

Supplement: S5 Fig — (A) Representative pictures of H/E- stained sections from the back skin of 10-week-old female mice. Scale bar: 200 μm. (B) Flow cytometry analysis of dissociated single cells from the back skin epidermis or dermis of tg/wt and tg/ko mice for the quantification of different immune cell populations as indicated. Bars indicate mean ±SD. (PDF) [file pone.0187162.s005.pdf]
